# Supplementary material for: Fabrication of mm-Scale Complementary Split Ring Resonators, for Potential Application as Water Pollution Sensors
Source: Materials (Basel). 2023 Jul 27;16(15):5290. doi: 10.3390/ma16155290 (PMC10420294; doi:10.3390/ma16155290)
Supplement: Supplementary file 1 [file materials-16-05290-s001.zip › materials-2464144-supplementary.pdf]

Fabrication of mm-scale complementary split ring resonators, for potential application as water pollution sensors

Zacharias Viskadourakis, George Fanourakis, Evangelos Tamiolakis, Anna Theodosi, Klytaimnistra Katsara, Nikolaos Rafael Vrithias, Odysseas Tsilipakos and George Kenanakis

Supplementary Material

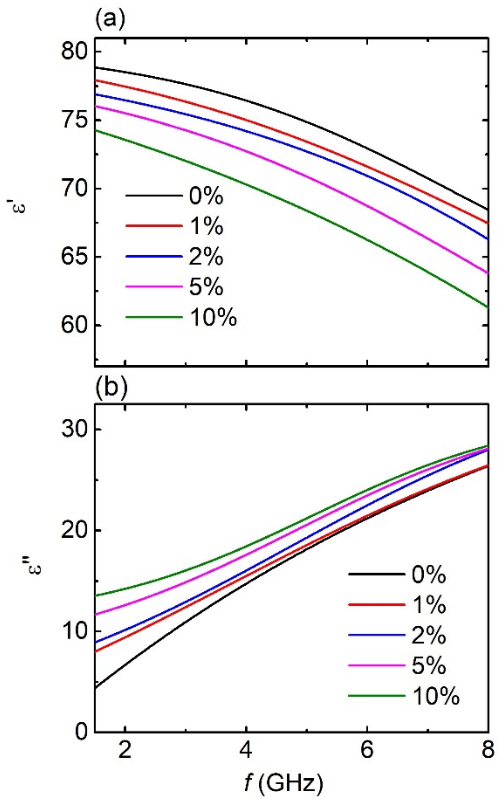

Figure S1: Dielectric permittivity vs. frequency for aqueous solutions containing fertilizer, in various concentrations, i.e., 10% wt. (green line), 5% wt. (magenda line), 2% wt. (blue line), 1% wt. (red line) and 0% (black line – pure water). **(a)** real part and **(b)** imaginary part. Dielectric permittivity has been measured using the N1501A Dielectric Probe Kit (Keysight, California, USA), in combination with the N1500A Materials Measurement Suite (Keysight, California, USA), for data analysis.

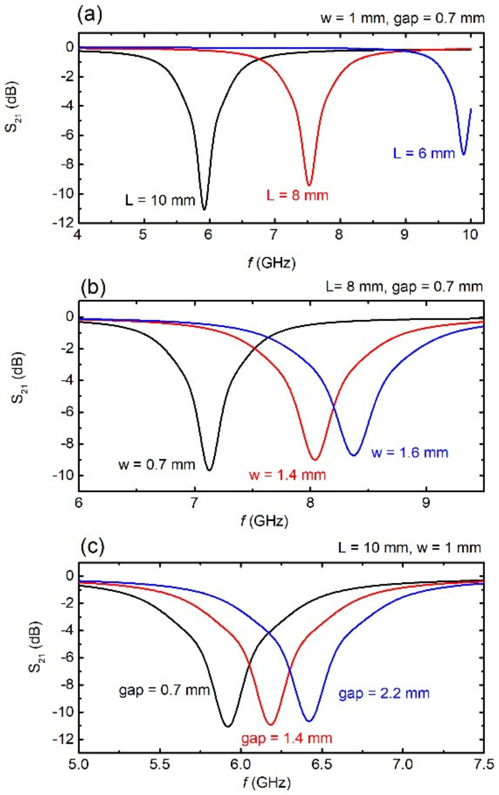

Figure S2: S21 vs. *f* curves, for rectangular complementary split ring resonators, as determined from theoretical simulations. (a) Length dependence **(b)** line width dependence **(c)** gap dependence

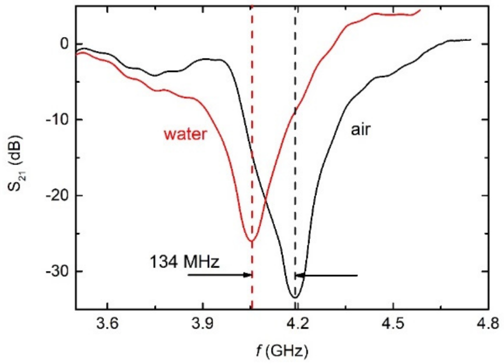

Figure S3: S21 vs. *f*, for the C1 sample with (red solid line) and without (black solid line) the presence of water into the engraved area.

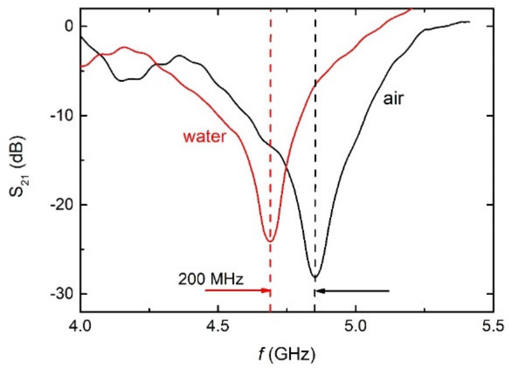

Figure S4: S_{21} vs. *f*, for the C3 sample with (red solid line) and without (black solid line) the presence of water into the engraved area.

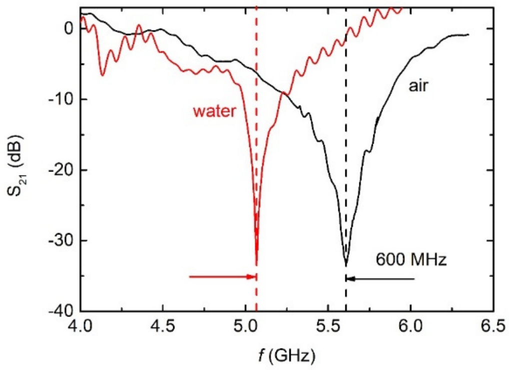

Figure S5: S_{21} vs. *f*, for the C4 sample with (red solid line) and without (black solid line) the presence of water into the engraved area.

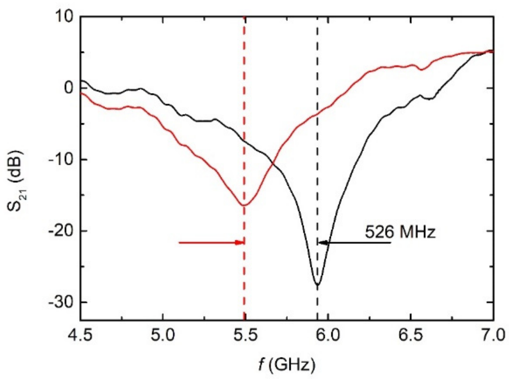

Figure S6: S_{21} vs. *f*, for the C6 sample with (red solid line) and without (black solid line) the presence of water into the engraved area.

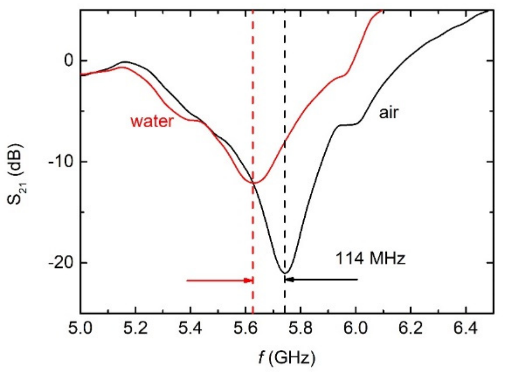

Figure S7: S_{21} vs. *f*, for the C10 sample with (red solid line) and without (black solid line) the presence of water into the engraved area.

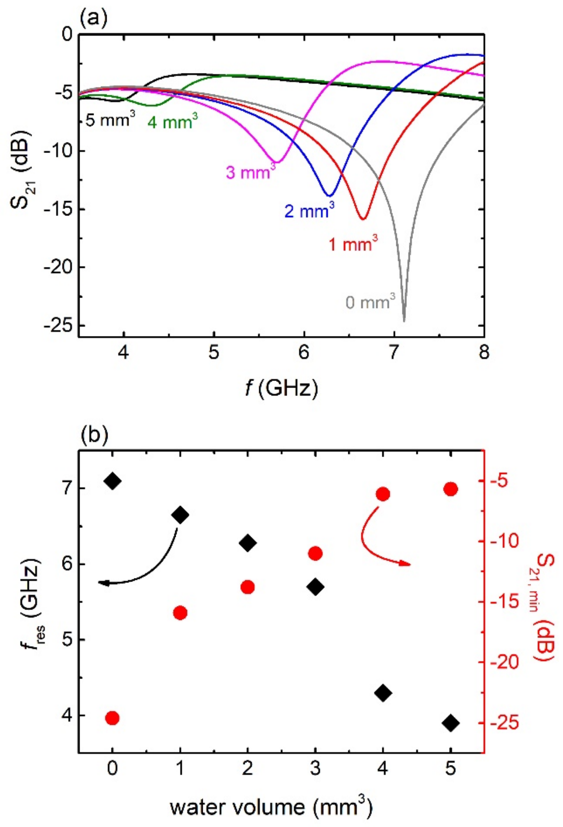

Figure S8: Theoretical simulations, regarding the injection of various water quantities into the engraved area of the CSRR. Dimension of the CSRR: L=7mm, w=1mm, gap=1.2mm, engraved depth = 0.2mm. The metal coating is 35 μm. (a) S_{21} vs f curves **(b)** Resonance frequency vs. water volume (black symbols) and intensity vs. water volume (red symbols), as extracted from panel (a).

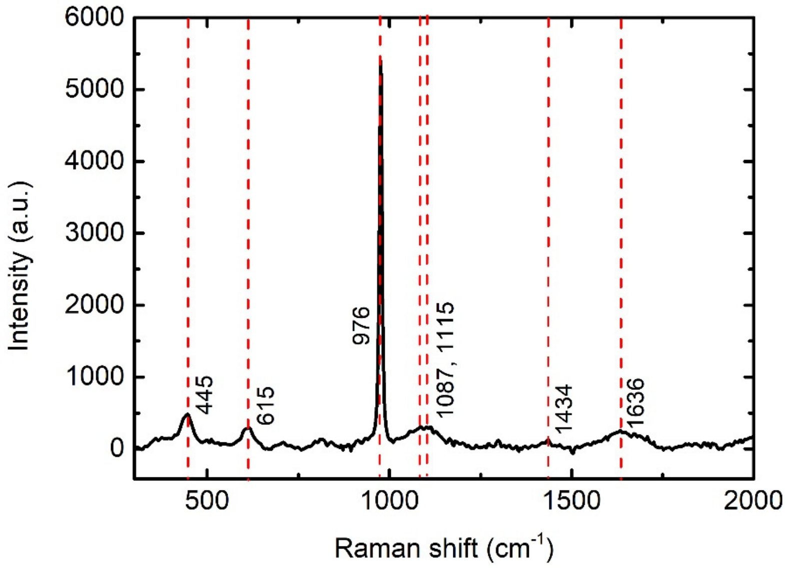

Figure S9: Raman spectrum of the ammonium sulphate [$(NH_4)_2SO_4$], the main ingredient of the fertilizer used in aqueous solutions, studied in the main text. All peaks observed can be ascribed to the intra-molecular vibrations of the ammonium sulphate. .

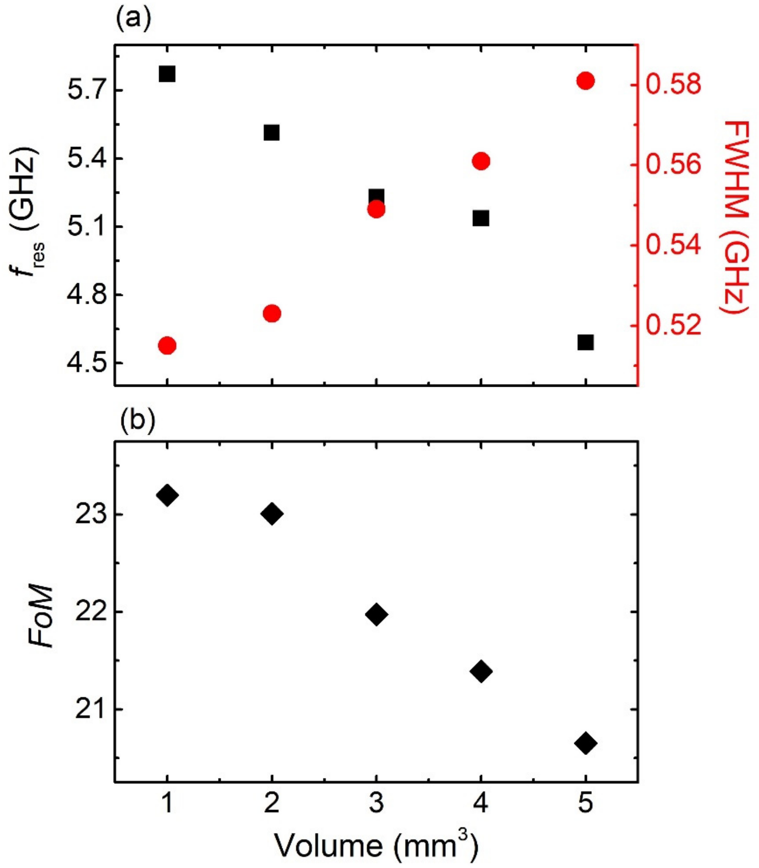

Figure S10: (a). Resonance frequency (black rectangles), and corresponding FWHM (red circles), as a function of water volume. Data are extracted from Fig. 4a of the main text **(b). Calculated FoM as a function of volume**. A clear lope change is observed above ~2mm^3, suggesting the inferior performance of the MS, above such limit.

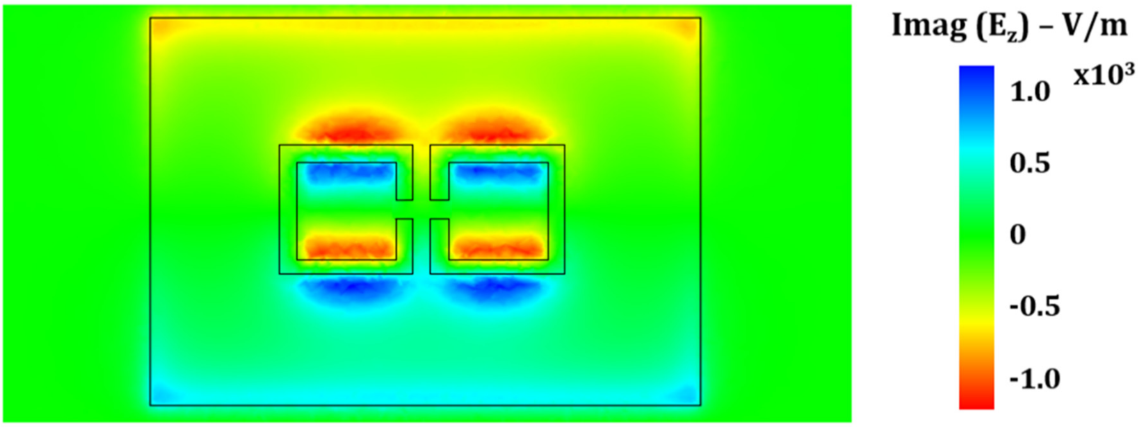

Figure S11: Electric field distribution (imaginary part of E_z component) at the resonance frequency of 7.05GHz without the presence of water. Dimensions of the CSRR: L=7mm, w=1mm, gap=1.2mm, engraved depth=0.2mm. The metal coating is 35μm.

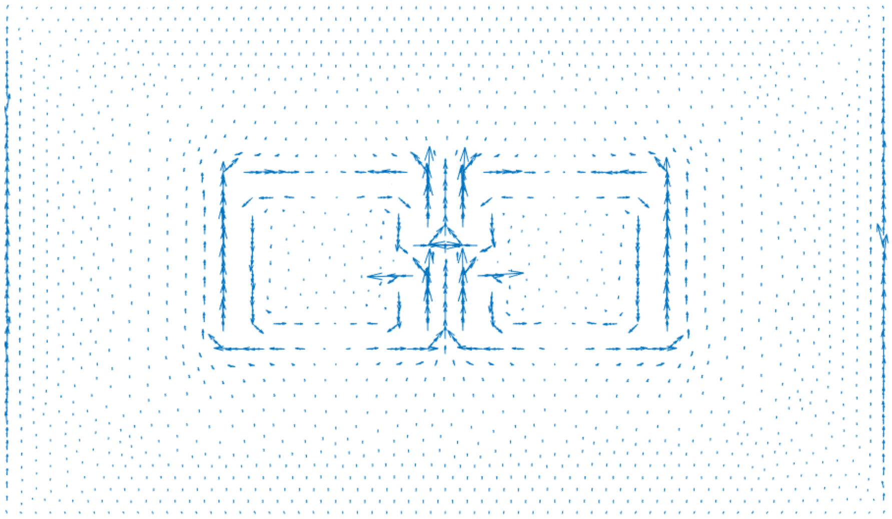

Figure S12: Current density distribution (J_x and J_y components) at the resonance frequency of 7.05GHz without the presence of water. Dimensions of the CSRR: L=7mm, w=1mm, gap=1.2mm, engraved depth=0.2mm. The metal coating is 35μm.
